# The risk factors of the 72-h unscheduled return visit admission to emergency department in adults below 50 years old

**DOI:** 10.1186/s40001-023-01317-x

**Published:** 2023-09-27

**Authors:** Chia-Lung Kao, Chia-Chang Chuang, Chi-Yuan Hwang, Chung-Hsun Lee, Po-Chang Huang, Ming-Yuan Hong, Chih-Hsien Chi

**Affiliations:** Department of Emergency Medicine, National Cheng Kung University Hospital, College of Medicine, National Cheng Kung University, No.138, Sheng Li Road, Tainan, 704 Taiwan

**Keywords:** Charlson comorbidity index, Emergency department, Unscheduled revisit, Admission

## Abstract

**Background:**

An unscheduled return visit (URV) to the emergency department (ED) within 72-h is an indicator of ED performance. An unscheduled return revisit (URV) within 72-h was used to monitor adverse events and medical errors in a hospital quality improvement program. The study explores the potential factors that contribute to URV to the ED within 72-h and the unscheduled return revisit admission (URVA) in adults below 50 years old.

**Methods:**

The case–control study enrolled 9483 URV patients during 2015–2020 in National Cheng-Kung University Hospital. URVA and URV non-admission (URVNA) patients were analyzed. The Gini impurity index was calculated by decision tree (DT) to split the variables capable of partitioning the groups into URVA and URVNA. Logistic regression is applied to calculate the odds ratio (OR) of candidate variables. The α level was set at 0.05.

**Results:**

Among patients under the age of 50, the percentage of females in URVNA was 55.05%, while in URVA it was 53.25%. Furthermore, the average age of URVA patients was 38.20 ± 8.10, which is higher than the average age of 35.19 ± 8.65 observed in URVNA. The Charlson Comorbidity Index (CCI) of the URVA patients (1.59 ± 1.00) was significantly higher than that of the URVNA patients (1.22 ± 0.64). The diastolic blood pressure (DBP) of the URVA patients was 85.29 ± 16.22, which was lower than that of the URVNA (82.89 ± 17.29). Severe triage of URVA patients is 21.1%, which is higher than the 9.7% of URVNA patients. The decision tree suggests that the factors associated with URVA are “severe triage,” “CCI higher than 2,” “DBP less than 86.5 mmHg,” and “age older than 34 years”. These risk factors were verified by logistic regression and the OR of CCI was 2.42 (1.50–3.90), the OR of age was 1.84 (1.50–2.27), the OR of DBP less than 86.5 was 0.71 (0.58–0.86), and the OR of severe triage was 2.35 (1.83–3.03).

**Conclusions:**

The results provide physicians with a reference for discharging patients and could help ED physicians reduce the cognitive burden associated with the diagnostic errors and stress.

## Background

The emergency department (ED) is at the forefront and must contend with crucial medical requirements. EDs are often crowded with patients waiting for medical treatment or admission. It is difficult to meet the emergency care needs of patients who crowd the ED [1, 2]. Because EDs are often crowded with patients in need of care, rapid discharge can reduce the burden on the ED. However, discharging patients without careful medical examination can compromise medical care quality and patient safety [3]. If the patient’s symptoms persist or recur, then patients will need to revisit the ED for medical help. An unscheduled return visit (URV) to the ED within 72-h is an indicator of ED performance. An URV within 72-h was used to monitor adverse events and medical errors in a hospital quality improvement program [3].

According to a previous study, 32.5% of unplanned return visits (URVs) within a 72-h timeframe were found to be avoidable [4]. These URVs can occur due to various factors, including medical errors, patient deterioration, non-adherence to treatment, and complicated symptoms. Previous research has highlighted the impact of medical errors on patient safety and healthcare costs [5]. Furthermore, studies have reported that URVs can be influenced by patient-based and illness-based factors [6, 7]. It has also been observed that URV patients tend to have a higher admission rate compared to those who do not experience a return visit [5, 8, 9]. Therefore, the rate of 72-h URVs serves as an indicator of the quality of hospital service. Understanding the potential risks associated with URV patients is crucial for improving medical care and enhancing the overall quality of hospital services [10]. Additionally, a 72-h URV not only leads to the waste of medical resources but also increases medical care costs [11, 12]. Lawsuits associated with URVs can also disrupt the medical system. Factors, such as age, triage, clinical division, and health education, prior to discharge have been identified as contributing to a higher URV rate. Elderly URV patients, in particular, are at a higher risk due to their medical complexity [13].

Extensive exploration and verification of the characteristics of 72-h URVs have been conducted in numerous studies, with comorbidity assessment, such as the Charlson Comorbidity Index (CCI), being utilized to predict the 30-day mortality rate for elderly patients visiting the emergency department (ED) [14, 15]. Additionally, a higher CCI score has been associated with admission, transfer, or death in the ED [16]. Existing evidence strongly supports the notion that elderly patients with a high CCI are highly prone to revisiting the ED within a 72-h timeframe.

However, there remains a scarcity of research focusing on URV patients within the working age population. The working age demographic is typically defined as individuals between 15 and 64 years old, as outlined by the Organization for Economic Co-operation and Development (OECD). In the context of Taiwan, data from the National Development Council's report reveal that over 76.7% of individuals under the age of 50 are actively participating in the labor force [17]. This particular age group constitutes the primary driving force behind gross domestic product (GDP) growth and assumes vital roles in supporting both their families and the overall society. Reducing the occurrence of URVs among these productive individuals would be advantageous not only to their families but also to the country as a whole. While significant research has been conducted to identify the factors influencing URVs in the elderly population, the same level of attention has not been given to younger URV patients. Consequently, the aim of this study is to explore the potential factors that contribute to the occurrence of 72-h URVs and subsequent admissions in patients below the age of 50. By addressing this research gap, we can gain a more comprehensive understanding of the factors influencing URVs in this specific age group and work toward reducing their occurrence effectively.

## Methods

### Study design and setting

We conducted the case–control study by reviewing the 72-h URV patients in National Cheng Kung University Hospital (NCKUH), which was approved by the Ethics Review Board of NCKUH. The case group was determined according to the 72-h URV patients whom admitted to the hospital. The control group was determined according to the 72-h URV patients whom did not admitted to the hospital.

### Inclusion and exclusion criteria

The 72-h URV patients visit to NCKUH ED during January 1 of 2015 to March 31 of 2020 were included. The URV patients less than 18 years old and the trauma patients were excluded.

### Variables definition

Data collected from medical records included major diagnostic findings, vital signs (blood pressure, heart rate, etc.).

Charlson Comorbidity Index [18], originally consisting of 19 items corresponding to different comorbid conditions, was applied to different populations as a prognostic measure to predict mortality in longitudinal studies [15]. A higher score on the index indicates a greater likelihood of predicted outcomes leading to mortality, with a score of zero indicating the absence of any comorbidities.

Rapid Acute Physiology Score (RAPS) [19], was specifically developed as a severity scale for critical care transports. RAPS is a condensed adaptation of the Acute Physiology and Chronic Health Evaluation (APACHE-II), focusing on parameters that are readily accessible for all transported patients. It includes measurements, such as pulse, blood pressure, respiratory rate, and the Glasgow Coma Scale.

Sequential Organ Failure Assessment (qSOFA) [20], designed for patients not in the intensive care unit (ICU), serves as a tool to identify high-risk individuals. It helps to flag patients who may require immediate attention or escalation of care.

Shock Index [21], is calculated by dividing the heart rate (HR) by the systolic blood pressure (SBP).

5-level Taiwan Triage and Acuity Scale (TTAS) triage system used in Taiwan to categorize emergency patients based on their vital signs and overall clinical status. Ranging from the most severe (Level 1) to the least severe (Level 5), level 1 to level 2 triage was classified as severe triage [22].

Length of stay denotes the duration of patients' stays in the ED, providing insights into the time they spend receiving medical care.

Age is an additional factor used to calculate CCI, and people’s age younger than 50 with a CCI less than 2 was classified as younger and having low comorbidity [23]. In order to compare the factors associated with admission in different age, the age was stratified as the subgroup of age below 50, 50–60, 60–70, 70–80, and age above 80.

Additionally, other variables were considered, such as discharge/admission, rotation, weekday/weekend, causes related to revisit (symptoms not relief, recurrence, complications, new clinical problem, misdiagnosis, improper medical disposing, disposing sequela, adverse drug reaction, diagnosis certification, etc.) and the record of discharge. The primary outcome is the 72-h URVA (patients’ unscheduled revisit the ED with admission in 72-h). We compared the differences in the potential risk factors associated with 72-h URVA between the two groups. The patients were further subdivided by the age of 50 on the basis of the CCI definition.

### Statistical analysis

The categorical variables are presented as percentages (%) and were tested by the χ2-test, continuous variables are presented as the mean ± standard deviation and were tested with Student’s t test. To investigate the potential predictive factors for patients with a low CCI and a low rate of URVA, we therefore focused on the group of patients younger than 50 years old. To identify potential risk factors associated with unscheduled revisits to the emergency department, we employed a decision tree (DT) methodology. The decision tree utilized the Gini impurity index, a measure of node impurity, to effectively split the variables and partition the study participants into two distinct groups: URVA and URVNA. Utilizing this machine learning technique, we aimed to uncover the key variables that contribute to the differentiation between these groups. To conduct the decision tree analysis, we utilized two R packages, “rpart” and “partykit”. The DT model was verified by logistic regression to calculate the odds ratio (OR) of candidate variables. The α level was set at 0.05. We applied R (ver. 3.6.2) to conduct the data analysis.

## Results

### Demographic

**Fig. 1:**
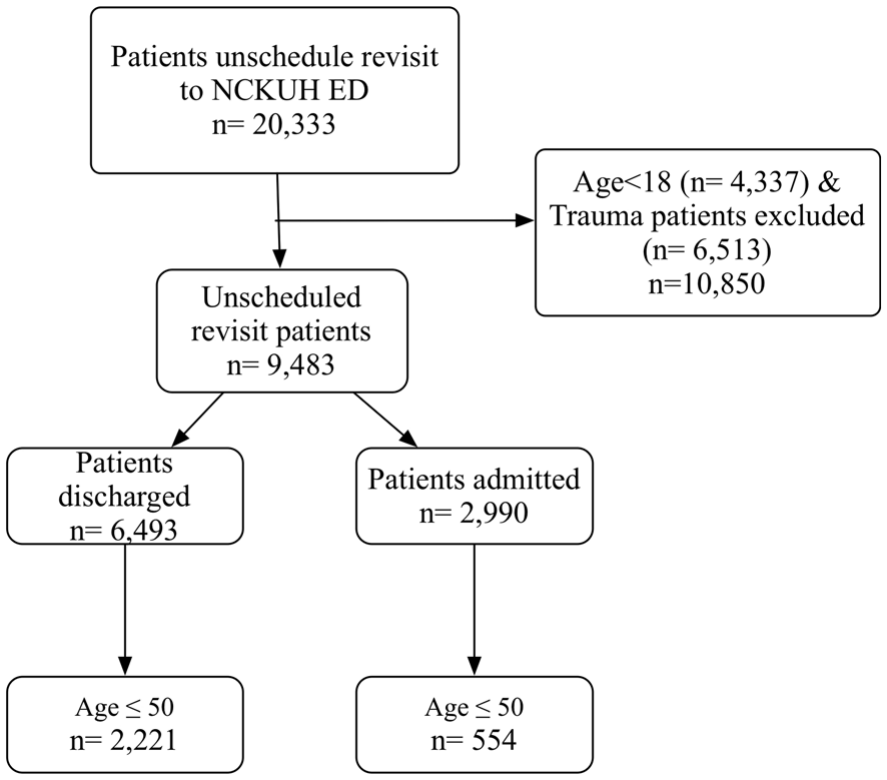
Flowchart of participant selection

Figure 1 is the flow chart of enrolling the study participants. The URV patients were further subdivided according to the age category and admission/discharge on the revisit determination (Table 1).

**Table 1:** Demographics of the 72-h URVNA and URVA patients

	Age ≤ 50			50 ~ 60		*p *value	60 ~ 70		*p *value	70 ~ 80		*p *value	Age ≥ 80		
	URVNA	URVA	*p *value	URVNA	URVA		URVNA	URVA		URVNA	URVA		URVNA	URVA	*p *value
*n*	2221	554		1020	402		1349	727		968	599		708	935	
Sex (%)															
Male	998 (44.93)	259 (46.75)	0.47	573 (56.21)	255 (63.4)	0.01	648 (48.02)	397 (54.61)	< 0.001	465 (48.07)	354 (59.12)	< 0.001	368 (51.97)	457 (48.88)	0.23
Female	1223 (55.07)	295 (53.25)	0.47	447 (43.89)	147 (36.6)	0.01	701 (52.98)	330 (45.39)	0.004	503 (51.93)	245 (40.88)	< 0.001	340 (48.03)	478 (51.12)	0.23
Length of stay	4.33 (6.07)	6.63 (8.31)	< 0.001	5.43 (7.92)	7.76 (10.01)	< 0.001	5.57 (7.30)	7.93 (8.54)	< 0.001	6.46 (8.11)	8.26 (8.80)	< 0.001	9.12 (9.19)	6.27 (7.37)	< 0.001
Triage (%)															
Severe	217 (9.77)	117 (21.12)	< 0.001	151 (14.80)	108 (26.87)	< 0.001	217 (16.09)	239 (32.87)	< 0.001	190 (19.63)	203 (33.89)	< 0.001	176 (18.82)	287 (40.54)	0.01
Non-severe	2004 (90.23)	437 (78.88)	< 0.001	869 (85.20)	294 (73.13)	< 0.001	1132 (83.91)	488 (67.13)	< 0.001	778 (80.37)	396 (66.11)	< 0.001	532 (81.18)	748 (59.46)	0.02
Vital Sign (mean (SD))															
Temp	37.02 (2.31)	37.22 (1.95)	0.04	36.78 (1.85)	36.99 (2.15)	0.08	36.75 (1.66)	37.02 (2.27)	0.01	36.70 (1.85)	37.13 (1.92)	< 0.001	36.77 (1.43)	37.11 (0.96)	< 0.001
Breath	19.12 (3.13)	19.50 (4.07)	0.04	19.05 (2.32)	19.41 (3.25)	0.04	19.13 (2.29)	19.91 (4.25)	< 0.001	19.21 (2.55)	19.75 (3.06)	< 0.001	19.49 (3.53)	20.48 (4.51)	< 0.001
SBP	132.46 (22.87)	129.92 (23.81)	0.02	143.75 (27.32)	138.25 (27.94)	< 0.001	146.64 (28.23)	139.80 (30.99)	< 0.001	148.42 (29.57)	139.10 (27.73)	< 0.001	146.45 (30.43)	141.65 (30.67)	0.001
DBP	85.29 (16.22)	82.89 (17.29)	0.003	89.66 (17.38)	86.39 (17.52)	0.001	87.30 (16.54)	83.91 (20.94)	< 0.001	83.53 (16.66)	78.59 (16.89)	< 0.001	77.46 (17.25)	76.72 (17.60)	0.40
Pulse	95.78 (20.66)	100.98 (21.78)	< 0.001	92.01 (19.41)	97.88 (22.38)	< 0.001	90.27 (19.63)	97.36 (23.42)	< 0.001	87.23 (18.05)	92.25 (21.48)	< 0.001	85.28 (18.83)	88.86 (21.18)	< 0.001
Index Score (mean (SD))															
RAPS	2.33 (1.45)	2.53 (1.56)	< 0.001	2.69 (1.53)	2.77 (1.65)	0.39	2.60 (1.53)	2.83 (1.83)	0.003	2.54 (1.52)	2.55 (1.65)	0.78	2.41 (1.60)	2.69 (1.74)	< 0.001
qSOFA	1.09 (0.32)	1.17 (0.42)	< 0.001	1.11 (0.33)	1.20 (0.47)	< 0.001	1.11 (0.34)	1.24 (0.52)	< 0.001	1.14 (0.39)	1.28 (0.51)	< 0.001	1.20 (0.45)	1.41 (0.63)	< 0.001
CCI	1.22 (0.64)	1.59 (1.00)	< 0.001	2.74 (1.15)	3.36 (1.35)	< 0.001	4.09 (1.32)	4.64 (1.45)	< 0.001	5.35 (1.36)	5.77 (1.43)	< 0.001	6.47 (1.49)	6.79 (1.59)	< 0.001
GCS	14.87 (1.10)	14.80 (1.24)	0.22	14.87 (0.95)	14.76 (1.27)	0.13	14.88 (0.84)	14.72 (1.37)	0.003	14.81 (1.16)	14.68 (1.32)	0.06	14.75 (1.12)	14.29 (1.90)	< 0.001
Shock Index (HR/SBP)	0.74 (0.18)	0.80 (0.21)	< 0.001	0.66 (0.18)	0.74 (0.23)	< 0.001	0.64 (0.19)	0.73 (0.24)	< 0.001	0.61 (0.18)	0.68 (0.20)	< 0.001	0.61 (0.19)	0.65 (0.21)	< 0.001
Comorbidity (%)															
MI	2 (0.09)	3 (0.54)	0.09	4 (0.39)	10 (2.49)	< 0.001	8 (0.59)	16 (2.20)	0.002	5 (0.52)	16 (2.67)	< 0.001	7 (0.75)	14 (1.98)	0.49
CHF	14 (0.63)	16 (2.89)	< 0.001	14 (1.37)	11 (2.74)	0.12	44 (3.26)	45 (6.19)	0.002	54 (5.58)	51 (8.51)	0.03	70 (7.49)	97 (13.70)	0.81
PVD	2 (0.09)	0 (0.00)	NA	1 (0.10)	1 (0.25)	NA	4 (0.30)	4 (0.55)	0.60	0 (0.00)	3 (0.50)	NA	4 (0.43)	5 (0.71)	1.00
CVA	10 (0.45)	5 (0.90)	0.33	18 (1.76)	11 (2.74)	0.34	37 (2.74)	32 (4.40)	0.06	42 (4.34)	48 (8.01)	0.003	85 (9.09)	66 (9.32)	< 0.001
Dementia	0 (0.00)	0 (0.00)	NA	0 (0.00)	0 (0.00)	NA	3 (0.22)	0 (0.00)	NA	6 (0.62)	8 (1.34)	0.24	34 (3.64)	39 (5.51)	0.62
COPD	6 (0.27)	1 (0.18)	NA	12 (1.18)	5 (1.24)	1.00	30 (2.22)	31 (4.26)	0.01	38 (3.93)	31 (5.18)	0.30	42 (4.49)	54 (7.63)	0.98
CTD	20 (0.90)	27 (4.87)	< 0.001	6 (0.59)	1 (0.25)	NA	13 (0.96)	15 (2.06)	0.06	13 (1.34)	5 (0.83)	0.50	6 (0.64)	12 (1.69)	0.55
PUD	27 (1.22)	3 (0.54)	0.25	16 (1.57)	14 (3.48)	0.04	49 (3.63)	23 (3.16)	0.67	24 (2.48)	19 (3.17)	0.51	20 (2.14)	27 (3.81)	1.00
Liver disease	24 (1.08)	20 (3.61)	< 0.001	40 (3.92)	30 (7.46)	0.008	66 (4.89)	53 (7.29)	0.03	44 (4.55)	32 (5.34)	0.55	22 (2.35)	26 (3.67)	0.81
DM	113 (5.09)	38 (6.86)	0.12	127 (12.45)	87 (21.64)	< 0.001	282 (20.90)	227 (31.22)	< 0.001	301 (31.10)	238 (39.73)	< 0.001	273 (29.20)	259 (36.58)	< 0.001
CKD	24 (1.08)	12 (2.17)	0.07	49 (4.80)	24 (5.97)	0.44	88 (6.52)	75 (10.32)	0.003	121 (12.50)	97 (16.19)	0.05	199 (21.28)	158 (22.32)	< 0.001
Tumor	75 (3.38)	66 (11.91)	< 0.001	168 (16.47)	132 (32.84)	< 0.001	306 (22.68)	243 (33.43)	< 0.001	223 (23.04)	171 (28.55)	0.02	183 (19.57)	147 (20.76)	< 0.001
Leukemia	7 (0.32)	5 (0.90)	0.13	0 (0.00)	2 (0.50)	NA	0 (0.00)	4 (0.55)	NA	1 (0.10)	2 (0.33)	0.67	0 (0.00)	2 (0.28)	NA
Lymphoma	2 (0.09)	0 (0.00)	NA	5 (0.49)	4 (1.00)	0.48	6 (0.44)	2 (0.28)	0.82	1 (0.10)	4 (0.67)	0.14	1 (0.11)	6 (0.85)	0.25
AIDS	14 (0.63)	15 (2.71)	< 0.001	2 (0.20)	0 (0.00)	NA	2 (0.15)	1 (0.14)	1.00	2 (0.21)	2 (0.33)	1.00	0 (0.00)	0 (0.00)	NA

*Temp* temperature, *SBP* systolic blood pressure, *DBP* diastolic blood pressure, *RAPS* Rapid Acute Physiology Score, *qSOFA* Sequential Organ Failure Assessment, *CCI* Charlson Comorbidity Index, *PVD* peripheral artery disease, *GCS* Glasgow Coma Scale, *MI* myocardial infarction, *CHF* congestive heart failure, *PVD* peripheral vascular disease, *CVA* cerebrovascular accident, *COPD* chronic obstructive pulmonary disease, *CTD* connective tissue disease, *PUD* peptic ulcer disease, *DM* diabetes mellitus, *CKD* chronic kidney disease, *AIDS* acquired immunodeficiency syndrome

Consistently, patients younger than 50 years old had the lowest CCI score and the lowest URVA rate compared to the other age-stratified subgroups. The rate of URVA in each age-stratified subgroup of age below 50, 50–60, 60–70, 70–80, and age above 80 was 19.9%, 28.3%, 35.0%, 38.2%, and 56.9%, respectively. We subdivided the patients according to age for further comparison because we found that 19.9% of the younger and low comorbidity URV patients were admitted to the hospital (Table 1). In the category of patients younger than 50 years old, the proportion of females (53.2%) was higher than the proportion of males. The mean length of stay of the URVA patients was 6.63 ± 8.31 h, which was longer than that of the URV without admission (URVNA) patients (4.33 ± 6.07 h). A total of 78.9% of the 554 URVA patients were classified as severe triage. The RAPS of URVA is 2.53 ± 1.56, which is higher than the 2.33 ± 1.45 of URVNA. The qSOFA of URVA was 1.17 ± 0.42, which was higher than the 1.09 ± 0.32 of URVNA patients. The URVA patients had a higher CCI (1.59 ± 1.00) than the URVNA patients (1.22 ± 0.64).

The URVA patients had a higher Shock Index (0.80 ± 0.21) than the URVNA patients (0.74 ± 0.18). Comparing the comorbidities of URVA and URVNA patients, the top three comorbidities were solid tumor (11.9% vs. 3.4%), DM (6.9% vs. 5.1%), and CTD (4.9% vs. 0.9%).

### Revisit reasons of the URV patients age below 50 years old within 72-h

Comparing the revisit reasons between the URVNA and URVA patients, “progression of disease” was the main cause (34.5%) of the URVA group, which was higher than that of the URVNA patients (27.1%). The “recurrent disease progress” in URVA patients was 10.5%, which was lower than that of URVNA patients (17.4%). The “New problem” percentage is not different between URVA and URVNA. The revisit reason of “Complication” was 9.0% in the URVA group, higher than the 4.4% of the URVNA group. The main cause of 72-h URVA is “Illness-related”. The percentage of “Misdiagnosis” in URVA was 2.9%, which was higher than the percentage of URVNA (1.6%) (Table 2).

**Table 2:** Reasons for the URV age below 50 years old within 72-h compared by admission versus discharge

Classification	Sub classification	URVNA (*n* = 2221)	URVA (*n* = 554)	*p* value
Illness-related	Progression of disease	604 (27.19)	191 (34.48)	< 0.001
Illness-related	Recurrent disease progress	387 (17.42)	58 (10.47)	< 0.001
Illness-related	Complication	99 (4.46)	50 (9.03)	< 0.001
Illness-related	New problem	404 (18.19)	103 (18.59)	0.83
Physician-related	Misdiagnosis	28 (1.26)	13 (2.35)	0.09
Physician-related	Failure of reassessment	1 (0.04)	1 (0.18)	0.85
Physician-related	Treatment error	1 (0.04)	0 (0.0)	NA
Physician-related	Drug side effect	6 (0.72)	0 (0.0)	NA
Patient-based	Social issue	193 (8.69)	26 (4.69)	0.003
Health care system-related	Hospital issue	20 (0.90)	1 (0.18)	0.14
Others	Others	26 (1.17)	6 (1.08)	1.00

Tested by Chi-square test

### Diagnosis of the URV patients younger than 50 years old

Of the 554 URVA patients, abdominal pain was the major diagnostic factor (21.7%), followed by fever (19.1%), anorexia (5.2%), and dyspnea (4.5%). Of the 2221 URVNA patients, abdominal pain was the major diagnostic factor (23.1%), followed by fever (14.2%), anorexia (5.7%), dizziness (5.7%), and headache (5.2%). The percentage of fever and dyspnea in URVA patients was significantly higher than that in URVNA patients (Table 3).

**Table 3:** Diagnosis of URV patients younger than 50 years old

	URVNA (%)	URVA (%)	*p* value
	(*n* = 2221)	(*n* = 554)	
Abdominal pain	514 (23.14)	120 (21.67)	0.29
Fever	316 (14.13)	106 (19.13)	0.004
Anorexia	128 (5.76)	29 (5.23)	0.71
Dyspnea	49 (2.21)	25 (4.51)	0.004
Image Examination	112 (5.04)	23 (4.15)	0.46
Headache	115 (5.18)	19 (3.43)	0.11
Dizziness	127 (5.72)	19 (3.43)	0.04
Chest Pain	92 (4.14)	18 (3.25)	0.41
Examination results abnormal	4 (0.18)	14 (2.53)	< 0.001
Skin lesion	25 (1.13)	14 (2.53)	0.02
Palpitation	27 (1.22)	10 (1.81)	0.38
Upper respiratory infection	131 (5.90)	22 (3.97)	0.08
Lumbago	45 (2.03)	8 (1.44)	0.48
Tic	21 (0.95)	7 (1.26)	0.66
Diarrhea	36 (1.62)	7 (1.26)	0.68
Lower limb pain	28 (1.26)	6 (1.08)	0.91
Conscious change	4 (0.18)	6 (1.08)	0.005

### Potential factors of admission selected by decision tree and multivariate logistic regression

**Fig. 2:**
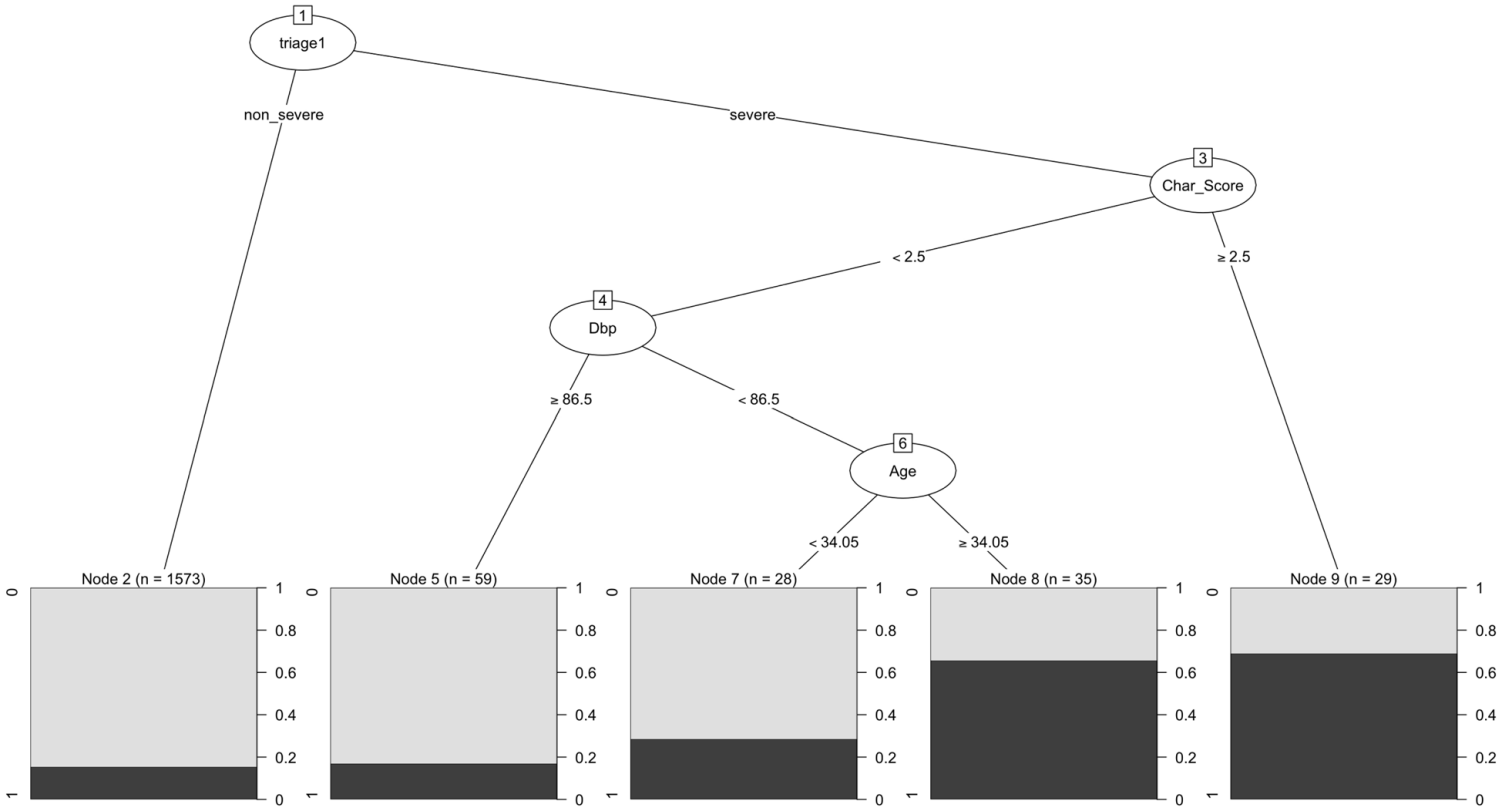
Decision tree that shows the filtering of the potential nodes that affect unplanned revisits in patients admitted to the hospital after the 2nd ER visit

The decision tree suggests potential factors to predict URVA patients, including “severe triage,” “CCI ≥ 2.5,” “DBP < 86.5 mmHg,” and “age ≥ 34.05” (Fig. 2). The cut point of CCI was determined as 3 and DBP was set as 87 to proceed the logistic regression.

The selected potential risk factors were verified by logistic regression (Table 4). With respect to the URVA patients, the OR of the patients with a CCI higher than 3 was 2.42 (95% confidence interval: 1.50—3.90). Patients older than 34 years had an OR of 1.84 (95% CI, 1.50—2.27) compared with patients younger than 34 years. The OR of females versus males was 0.94 (95% CI, 0.77–1.14). The OR of patients with a DBP higher than 87 mmHg was 0.71 (95% CI, 0.58–0.86) compared to the patients whose DBP was less than 87 mmHg. Patients with severe triage had an OR of 2.35 (95% CI, 1.83–3.03) compared to patients classified as non-severe triage.

**Table 4:** Logistic regression analysis for URVA patients who are younger than 50 years old

Variable	OR (95% CI)	AOR (95% CI)	Variable (cut point of the decision tree)	OR (95% CI)	AOR (95% CI)
CCI	1.69 (1.52–1.89)	1.51 (1.35–1.69)	CCI ≥ 3	3.01 (1.89–4.73)	2.42 (1.50–3.90)
Age	1.04 (1.03–1.05)	1.03 (1.02–1.04)	Age > 34	1.91 (1.56–2.32)	1.84 (1.50–2.27)
Sex (Female)	0.93 (0.77–1.12)	0.94 (0.77–1.15)	Sex (Female)	0.93 (0.77–1.12)	0.94 (0.77–1.14)
DBP	0.99 (0.99–1.00)	0.99 (0.98–0.99)	DBP ≥ 87	0.80 (0.66–0.97)	0.71 (0.58–0.86)
Triage (Severe)	2.48 (1.93–3.17)	2.07 (1.60–2.68)	Triage (Severe)	2.48 (1.93–3.17)	2.35 (1.83–3.03)

*CCI* Charlson Comorbidity Index, *DBP* diastolic blood pressure, *AOR* adjusted odds ratio

## Discussion

The study suggested that CCI ≥ 3, DBP ≤ 87, and age > 34 (Table 4) measured on the initial visit to the ED are potential risk factors associated with patients admitted to the hospital while they revisit the ED for 72-h.

The results showed that the length of stay for URVA patients was 6.63 h, which is longer than the 4.33 h of the URVNA patients. This means that URVA patients have more severe conditions than URVNA patients at the first ED visit, and the triage severity is consistent with the finding. The CCI of URVA patients was higher than that of URVNA patients, which indicates that comorbidity is a risk factor for admission to the hospital. In particular, CHF, CTD, liver disease, DM, and solid tumors were significantly different between the discharged and admitted patients. This finding is consistent with that of a previous study that reported that potentially avoidable return visits were more severe in ill patients [10].

In geriatric research, URV and admission (URVA) were positively correlated with higher CCI scores [24]. High CCI scores were associated with the URV of elderly patients, but patients younger than 50 years old with low CCI scores were not well documented. The study presented the potential factors contributing to URVA within 72-h in patients with a low CCI, since elderly URV patients were well documented and the factors were reported to be associated with comorbidities [25–27].

Revisits were primarily illness-related in 72.6% of the URVA patients, such as “progression of disease,” “recurrent disease progression,” “complication,” and “new problem.” The top 3 diagnoses in URVA patients were “fever,” “dyspnea,” and “dizziness.” The percentage was higher than that in a previous study conducted in Spain, which reported that URV was due to illness in 61.1% of patients.^10^

A study analyzed the characteristics of patients who revisited the ED within 48 h and reported that dyspnea was the most common chief complaint [28]. This finding is consistent with our study, which found 4.6% of URVA patients were diagnosed with dyspnea, which is higher than the 2.2% in the URVNA group.

The initial node in the decision tree is triage. A previous study also indicated that severe triage is a risk factor for URVA and has an OR of 2.1 (95% CI 1.3–3.2) [5]. A CCI score higher than 3 is another risk factor, and this finding is consistent with a study that reported that a CCI score higher than 2 was associated with a higher admission rate [29]. A CCI score more than 3, a DBP less than 86.5, and an age older than 34 are secondary risk factors. The value of the “CCI”, “DBP”, and “Age” assessed at the first visit to the ED could be applied to predict whether the patients would be admitted to the hospital on the second visit to the ER. There is a worry that the occurrence of New problem as the causes for the URV within 72-h were approximately 20% in both groups (Table 2). The average age of URVA patients was 38.25, which is higher than the average age of 35.47 in URVNA. Additionally, the CCI value for URVA was 0.42, indicating a higher severity level compared to the CCI value of 0.23 observed in URVNA. Consequently, the decision tree might overestimate the nodes due to the Age and CCI values were significantly higher in the URVA group compared to the URVNA group. However, there was no significantly difference in the distribution of New problem between the two groups (18.59% in URVA versus 18.19% in URVNA), so the admission or discharge of patients was not correlated with the presence of New problem. Therefore, the concern that New problem may impact hospital admission would not bias the judgment of decision tree.

Low DBP could be a prognosis factor for hospital admission [30]. Our study also reported that a DBP less than 86.5 is a risk factor for URVA. Length of stay is a risk factor associated with URVA [31]. Our study also found that the length of stay in the URVA group was longer than that in the URVNA group, but the decision tree did not suggest length of stay as a risk factor. In addition, length of stay is an indicator of ED crowding, which can impact medical care quality. ED crowding implies that medical practitioners cannot meet the demands of patients in need of emergency care services, which contributes to poor quality of medical care services [32]. ED crowding might delay the delivery of necessary medical treatments in emergency conditions, thus leading to adverse patient health. ED crowding has been reported as a risk factor for 72-h URV [33].

We also analyzed the first ED visiting times and doctors’ shifts by logistic regression and found that both factors were significant in predicting revisit admission, but the decision tree ignored these factors. A previous study reported that URV percentage was not affected during the weekend or weekdays [34], which is also consistent with our findings.

The strength of the study is that we applied machine learning to screen the potential risk factors for URVA. These risk factors suggested by the decision tree were verified by logistic regression and were presented as an odds ratio.

The limitation of the study is that the factors that may potentially predict URV were not extensively collected. On the basis of machine learning, an adequate amount of variables is necessary for the decision tree, so that critical decision nodes can be suggested. The integrity of the variables is another weakness of the study. For example, we eliminated SpO_2_ because of missing data. Some of the records of the patients visiting the ED were missing because of the urgency of the emergency. There might exist a potential hospital-based selection bias in the study because the data were chart-reviewed from the NCKU hospital, a medical center in South Taiwan. A nationwide survey is suggested to obtain representative results in future.

## Conclusions

For patients younger than 50 years old, the logistic regression results suggested that CCI ≥ 3, DBP ≤ 87, and age > 34 measured on the initial visit to the ED are potential risk factors associated with patients admitted to the hospital while they revisit the ED for 72-h. The results provide physicians with a reference while discharging patients and might be helpful for ED physicians to release the cognitive load, which can result in diagnostic errors and stress [35].

## Data Availability

Not applicable.

## Abbreviations

CCI: Charlson Comorbidity Index
ED: Emergency department
URV: Unscheduled return visit
URVA: URV and admission
URVNA: URV and non-admission
OECD: The Organization for Economic Co-operation and Development
GDP: Gross domestic product
RAPS: Rapid Acute Physiology Score
qSOFA: Quick Sequential Organ Failure Assessment
TTAS: Taiwan Triage and Acuity Scale
DT: Decision tree
OR: Odds ratio
DBP: Diastolic blood pressure
